# Effect of Management System on Fecal Microbiota in Arabian Horses: Preliminary Results

**DOI:** 10.3390/vetsci12040309

**Published:** 2025-03-28

**Authors:** Maria Claudia Curadi, Flavio Vallone, Martina Tenuzzo, Angelo Gazzano, Valentina Gazzano, Fabio Macchioni, Claudia Vannini

**Affiliations:** 1 Department of Veterinary Sciences, University of Pisa, 56126 Pisa, Italy; maria.claudia.curadi@unipi.it (M.C.C.); f.vallone1@studenti.unipi.it (F.V.); angelo.gazzano@unipi.it (A.G.); fabio.macchioni@unipi.it (F.M.); 2 Department of Biology, Via Volta 4, 56126 Pisa, Italy; m.tenuzzo@studenti.unipi.it (M.T.); claudia.vannini@unipi.it (C.V.)

**Keywords:** Arabian horse, equine, fecal microbiota, GIT microbiota, microbiome, metabarcoding

## Abstract

The gut microbiota plays a basic role in maintaining the psychophysical health and well-being of horses. Understanding the complex interactions among microbial communities in relation to age, management, and health is a topic of growing interest. The equine microbiota, given its complexity, is subject to variations caused by internal or external stressors that can lead to metabolic problems and pathologies, i.e., obesity and laminitis. The aim of this study was to characterize the fecal microbiota of 12 purebred Arabian horses and to compare the microbial communities in two distinct management systems (Group 1 = box 22 h/day + paddock 2 h/day and Group 2 = paddock 24 h). Fecal samples were analyzed using high-throughput sequencing of 16S rRNA V3-V4 amplicons. The microbiota was predominantly composed of Firmicutes (32–53%) and Bacteroidetes (32–47.8%), with Ruminococcaceae emerging as the most prevalent bacterial family (10.9–24%). Comparisons of alpha and beta diversity revealed no statistically significant differences between the groups based on different management systems. This preliminary characterization contributes valuable data for understanding the equine fecal microbiota and lays the groundwork for future studies on the relationship among intestinal microbiota and equine health, performance, and management strategies.

## Simple Summary

Studies on the gut microbiota are currently of considerable importance in various research fields, both in human medicine and in veterinary medicine. With regard to equines, some studies are currently aimed at characterizing the intestinal microbial components and their stability, which can influence the psychophysical health and well-being in horses. The aim of this study was to analyze the fecal microbiota of 12 purebred Arabian horses by comparing microbial communities under two different management systems (Group 1 = box 22 h/day + paddock 2 h/day and Group 2 = paddock 24 h), while maintaining the same hay-based diet. This analysis also considers the growing interest in the relationship between breed and gut microbiota in equines. Fecal samples were analyzed using high-throughput sequencing of 16S rRNA V3-V4 amplicons. The management system did not influence the characteristics of the fecal microbiota in the subjects examined. The study of these characteristics in Arabian horses, with a strong aptitude for resistance exercises, could also contribute to the understanding of the relationships between intestinal microbiota and sports performance.

## 1. Introduction

The attention to the welfare of domestic animals has significantly increased in recent years, as demonstrated by the progressively increasing number of papers dedicated to this topic ([1,2,3,4,5]. Among domestic animals, horses play a particular role since they are considered a pet and a member of the family by a good number of horse owners [6]. The welfare of this species is, therefore, a subject of growing attention and study, particularly in light of the horse’s lifespan and recent experimental research on cognitive and memory abilities, regarding short- and long-term memory, including those in elderly horses [3,7,8,9].

Protecting the welfare of an animal species cannot ignore the knowledge of its natural behavior. Horses have evolved as grazing and browsing herbivores; in the wild, they graze most of the time, travelling long distances in groups, feeding on a wide variety of herbaceous species [10]. Traditional stabling, where horses spend most of the day confined to a stall, is the most common practice in Europe, USA and Canada; in relation to management and economic factors, the stabling system certainly offers the advantage of more continuous contact with the animals and a greater possibility of quickly providing care if necessary, as well as allowing the avoidance of negative interactions between the subjects or competitive behaviors that can lead to the risk of injuries especially in paddock [11,12].

This type of stabling has, however, a great influence on the welfare of the horse and prevents the animal from expressing important behaviors of its ethogram, such as socializing and foraging [13,14,15]. As a result, horses spend less time eating and more time standing, which has a negative impact on their physical and potentially mental health [16]. In addition to the evaluation of equine behavior, the welfare assessment is based on the detection of physiological and hormonal parameters, altered during stress [17,18].

Recently, the microbiota of the gastrointestinal tract (GIT) has been increasingly recognized as crucial for both human and animal health and welfare, since it plays a vital role in regulating essential biological processes such as metabolism, immune system function, protection against pathogens, and digestion [19,20,21,22,23].

Regarding the horse, focus has been placed on the composition of microbial communities, their relative ratio, and their impact on equine health and welfare, as well as their potential interactions with specific pathologies, behavioral traits [24,25,26] or athletic performance [27,28,29].

Variations in equine gut microbiota communities can be influenced by biotic and abiotic factors [30] such as diet, health status, breed, age, medical substances (e.g., anthelmintics), environmental conditions, social interactions, and stress-related factors [31,32,33,34]. Feral horses show an alteration in the microbiota diversity when compared to domesticated horses, highlighting an impoverishment of fecal microbiota in domestic subjects in the presence of probable triggers such as diet and antibiotic exposure [35,36]. Physical exertion have also been shown to alter host metabolism and intestinal microbiota, which may affect equine wellbeing and susceptibility to specific health issues [37]. Imbalances in gut microbiota can lead to digestive disorders like colics or hindgut acidosis, and may be associated with diseases such as colitis, equine metabolic syndrome, equine grass sickness, and laminitis, affecting health and performance [38,39,40,41,42,43].

Some authors have hypothesized the presence, in the large intestine of the horse, of a core bacterial community, in many cases identified as Operational Taxonomic Units (OTUs) or Amplicon Sequence Variants (ASVs), being present in all samples with a minimum relative abundance of 0.1% [39]. This core community would display a stable composition of microorganisms (composed of many OTUs in low abundance) which could partly explain the particular susceptibility of equines to digestive problems; in equines this core bacterial community appears to comprise a lower number of species than in ruminants, with a greater diversity in the right dorsal colon [39,44].

Firmicutes, a phylum of Gram-positive bacteria, are heavily involved in the fermentation of dietary fibers, leading to the production of short-chain fatty acids (SCFAs), such as butyrate [45,46]. These SCFAs are a crucial energy source for colonic epithelial cells and contribute significantly to maintaining gut health and integrity [47]. In horses, Firmicutes account for 40% to 90% of the total gut microbiota, representing the largest bacterial phylum [39]. On the other hand, Bacteroidetes, a phylum of Gram-negative bacteria, plays a central role in degrading complex carbohydrates. Their involvement in fermentative processes is crucial for generating SCFAs, which influence both energy metabolism and overall gut health [48,49]. The interaction between Firmicutes and Bacteroidetes is vital for maintaining microbial balance, or homeostasis, in the GIT; one of the most studied metrics for assessing this balance is the Firmicutes/Bacteroidetes (F/B) ratio, which is frequently explored as a potential biomarker for dysbiosis [50]. The F/B ratio is also associated with several pathological conditions, including obesity in humans [51] and intestinal diseases in horses [39].

Recent studies confirm to date that the equine gut microbiota can play a fundamental role in the physical and mental health of the performance horse. Stress and physical exertion can significantly impact the host’s metabolism and intestinal microbiota composition, with the hypothesis that certain bacteria are correlated with specific behavioral changes [25]. Some observational studies show the presence of differences in the fecal microbiota between cribbing and non-cribbing horse [26] and some Authors highlight an increase in the concentration of anaerobic bacteria in the equine colon in diets rich in starch and poor in fiber which constitute a nutritional stress for the horses that can produce changes in their behavior related to the greater presence of amylolytic bacteria [52].

The aim of this study was to characterize, through 16S rRNA amplicon metabarcoding, the bacterial diversity of fecal microbiota in purebred Arabian horses in relation to two diversified management systems.

## 2. Materials and Methods

### 2.1. Ethical Animal Research

The animal study protocol was reviewed and approved by the local animal care committee, Ethics Committee of University of Pisa, Italy, authorization n°17/2023, 19/04/2023: “Study on the fecal microbiota in Arabian horses”.

### 2.2. Animals

The study was designed to reflect the farm’s actual conditions, with animals housed under the stabling conditions described below.

Twelve clinically healthy Arabian horses, aged 2–25 years (BCS 5–6/9) without any behavioral alterations, were selected for this study. All the subjects were from the Arabian horse farm “Il Melograno” located in the province of Pistoia (Tuscany, Italy). The data set of horses enrolled for the experimental trial is reported in Table S1 of the supplementary data.

Six subjects (Group 1: C1, C2, C3, C4, C5, C6) were individually housed in boxes, each measuring 3.5 m x 3.5 m (half front door open, open at the back) with straw bedding, while six others (Group 2: C7, C8, C9, C10, C11, C12) were permanently and individually housed in paddocks. The stable horses had an average of two hours of individual daily outing in a sand paddock of approximately 400 m^2^ in size.

The paddocks, where the animals that live permanently outside were stabled, consisted of a non-grassy area, approximately 400 m^2^ in size, each equipped with run-in sheds and providing constant access to fresh water.

All subjects had received their last anthelmintic treatment six months before the trial. The diet for both groups of tested horses was the same and was based on hay (first-cut mixed-grass hay) (about 10 kg/subjects/day); the horses were fed hay 3 times a day, with a minimum supplement of grains (mix of 250 g of barley and oats: 2/3 oats and 1/3 barley) once a day. No bromatological analysis of the hay used was performed, and the horses did not receive any vitamin and mineral supplements.

### 2.3. Fecal Sampling

Twelve fecal samples were collected once in April 2023, with an external temperature range of 12–21 °C. All samples were collected on the same scheduled day, between 10:30 and 12:30 am. Rectal samples from each subject, approximately 200 g of feces from the center of the fecal ball, were collected with a gloved hand. Each biological sample was promptly placed into a sterile container to minimize environmental contamination and immediately refrigerated. After collection and transport to the laboratory for analysis, carried out within three hours of sampling, the samples were frozen in liquid nitrogen and stored in dedicated containers at −80 °C to preserve their integrity. At the same time, 5 g of each sample was stored in a 5 mL sterile vial to be analyzed for parasitological components. Throughout the entire process, utmost care was taken to maintain proper preservation of each sample, guaranteeing the quality and accuracy of subsequent analyses. This procedure limited the microbial contamination of the fecal samples in further analysis.

### 2.4. 16S rRNA Gene Metabarcoding

Fecal samples were handled and processed under sterile biological safety cabinets (Polaris 48, Steril Spa). Total genomic DNA was extracted from each fecal sample using the DNeasy Powersoil Kit (Qiagen, Hilden, Germany) following the manufacturer’s protocol, with an additional centrifuge step before loading onto the silica membrane. Subsequently, the concentration of extracted DNA was quantified using the Qubit 2.0 fluorometer and the Qubit dsDNA HS assay kit (Thermo Fisher Scientific, Waltham, MA, USA) before PCR. DNA amplification was performed under a sterile hood, with all equipment sterilized using UV radiation for a minimum of 4 h.

For this study, the V3-V4 region of the 16S rRNA gene was amplified using the following primers (5′–3′): forward—341F (CCTACGGGNGGCWGCAG) and reverse—785R (GACTACHVGGGTATCTAATCC) [53].

PCR reactions were prepared with the following components in a total volume of 50 µL: 10 µL of DNA (1 ng/µL); 1 µL (10 µM) of each primer; 25 µL of Kapa HotStart Ready MIX (Roche Diagnostics, Pleasenton, CA, USA); and 13 µL of dH2O. The amplification program was set as follows: 35 cycles (5 min at 95 °C; 30 s at 55 °C; 30 s at 72 °C) with a final extension phase of 5 min at 72 °C.

Subsequently, qualitative (1% agarose gel electrophoresis) and quantitative (Qubit 32.0) analyses of the amplified DNA were conducted before sending 30 µL of amplicons per sample for barcoding, pooling and sequencing on the Illumina NovaSeq platform in 2 × 250 bp paired-end format at IGA Technology Service laboratory (Udine, Italy). The obtained raw reads have been deposited in the European Nucleotide Archive (ENA) at EMBL-EBI under study accession number PRJEB80647.

### 2.5. Sequence Analysis

The software Quantitative Insights into Microbial Ecology (QIIME2, 2024.5 version, https://qiime2.org, accessed on 10 September 2024) (Quantitative Insights into Microbial Ecology) was used to analyze raw reads [54]. Demultiplexing, denoising, primer trimming, pair-end read merging, and de novo chimera removal were performed with the divisive amplicon denoising algorithm (DADA2) plugin [55]; ASVs with a total abundance of less than 10 were discarded before proceeding with downstream analyses. ASV representative sequences were aligned using MAFFT [56]; a phylogenetic tree was built with FASTTREE [57], manually checked, and, if necessary, additional chimeras were specifically removed from the dataset. Taxonomic assignment was performed using the Silva database [58]. A Naive Bayes classifier was trained, extracting the V3-V4 regions from SSU rRNA representative sequences (99% similarity clustered Operational Taxonomic Unit) as in Werner and colleagues [59]. ASVs identified as mitochondria, chloroplasts, unassigned, as well as all non-bacterial, were removed before further data processing.

### 2.6. Statistical Analysis

Using QIIME2, rarefaction curves were constructed for each sample with a sequencing depth of 277,108 reads to validate the performed screening. The same software was used to create a taxa bar plot of phyla and calculate alpha diversity and beta diversity using ASVs. For comparative analyses, a management system was considered. Alpha diversity was assessed by calculating the following indexes: number of sequence variants (ASVs), Shannon’s index (quantitative, non-phylogenetic index) for richness, and Pielou’s Evenness for evenness. Beta-diversity analyses were performed using Permanova and multivariate PCoA, testing various metrics: Bray–Curtis and Jaccard indexes for quantitative and qualitative data, respectively, and Uni-Frac distances, both weighted and unweighted, to assess the impact of phylogeny. The core microbial community for the analyzed samples was also assessed using QIIME2. As a minimum abundance threshold was already applied to all ASVs (see above), we included in the core microbiota ASVs retrieved in all samples, regardless of their relative abundance, in order not to skip essential microbes that were present in much lower quantities.

### 2.7. Parasitological Analysis

Coprological analyses of each subject kept in box and in paddock (Group 1, box: C1–C6; Group 2, paddock: C7–C12) were conducted on the stool samples to detect parasite oocysts, eggs, and larvae using the Mini-Flotac technique [60], with a solution of ZnCl2 (p.s. 1.200)

## 3. Results

### 3.1. Sequencing Depth and Data Screening

Data demonstrated that as the sequencing depth increased, the curve reached a plateau (Supplementary Figure S1), indicating that further increases in sequencing depth would not result in significant changes in the number of observed ASVs. This plateau suggests that the sequencing effort employed in this study was adequate to capture the full diversity of the fecal microbiota in the analyzed samples. Specifically, a total of 11,851 ASVs were identified, which provided a detailed quantitative measure of the variability present within the analyzed samples. The extensive range of ASVs confirms that the sequencing protocol was effective in reflecting the complexity and diversity of the microbial community within the fecal samples of purebred Arabian horses.

### 3.2. Microbiota Composition

**Figure 1:**
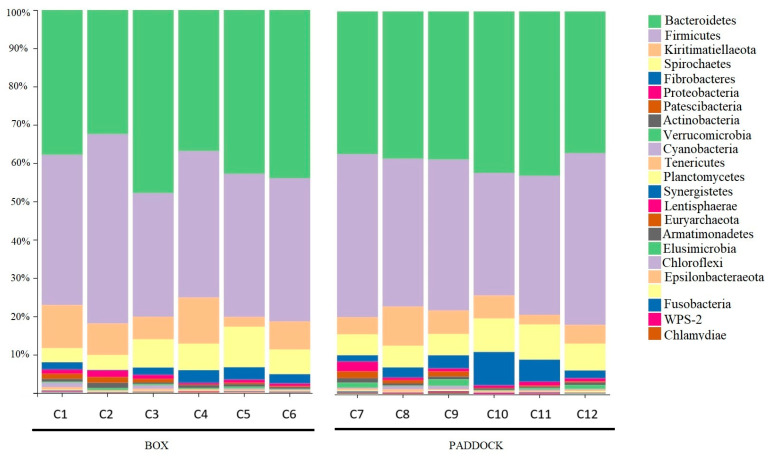
Relative frequency of phyla in the two different management systems (Group 1, box: C1–C6); (Group 2, paddock: C7–C12)

With the sequencing depth sufficient to capture microbial diversity, we next examined the composition of the fecal microbiota. The analysis revealed that the fecal microbiota in both groups (Group 1, box and Group 2, paddock) was predominantly composed of bacteria from the phyla Firmicutes, which constituted 32–53% of the microbiota across samples, and Bacteroidetes, representing 32–47.8% of the microbiota. Other notable phyla included Kiritimatiellaeota (2.5–12%), Spirochaetes (3.69–10.58%), Fibrobacteres (0.18–8.75%), and Proteobacteria (0.34–2.5%) (Figure 1).

At the family level, Ruminococcaceae emerged as the most prevalent, accounting for 10.9% to 24% of fecal microbiota. It was followed by Rickenellaceae (7.6–18%), Bacteroidales-p-251-o5 (2.07–17.3%), Lachnospiraceae (6.23–14.49%), and Prevotellaceae (5.79–11.80%). A core consisting of 186 ASVs retrieved in all Arabian horses was identified, with Clostridiales (Firmicutes) representing 68% (126 out of 186), mainly constituted by members of the Lachnospiraceae (22% of total core ASVs) and of the Ruminococcaceae (31% of total core ASVs) families. Additionally, a total of 371 genera were identified, reflecting the richness of the fecal microbiota at a finer taxonomic level. Among these were *Treponema* (3.61–10.48%), *Fibrobacter* (0.18–8.75%), *Phascolarctobacterium* (1.07–3.23%), and *Akkermansia* (0.18–1.6%).

### 3.3. Diversity Analysis

**Figure 2:**
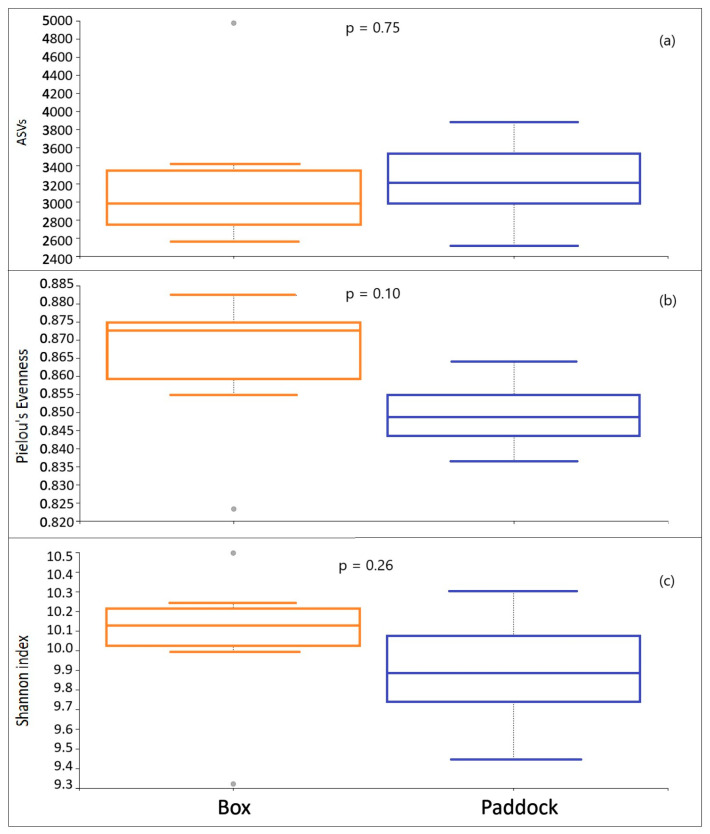
(**a**) Number of ASVs, (**b**) Pielou’s evenness, and (**c**) Shannon Diversity Index in the two groups of horses subjected to two different stabling methods (box and paddock).

To understand the variability within the microbiota, we calculated alpha diversity (number of ASVs, Shannon Diversity Index, and Pielou’s Evenness Index) across the two different management systems. Alpha diversity is a measure of how diverse a single sample is, usually considering the number of different species observed. The Shannon Index is an estimator for both species richness and evenness, but with a weight on the richness, while Pielou’s Evenness Index quantifies the evenness of species distribution. The Kruskal–Wallis test indicated that there were no statistically significant differences (*p* < 0.05) between the two management groups (Figure 2) for the three parameters considered.

**Figure 3:**
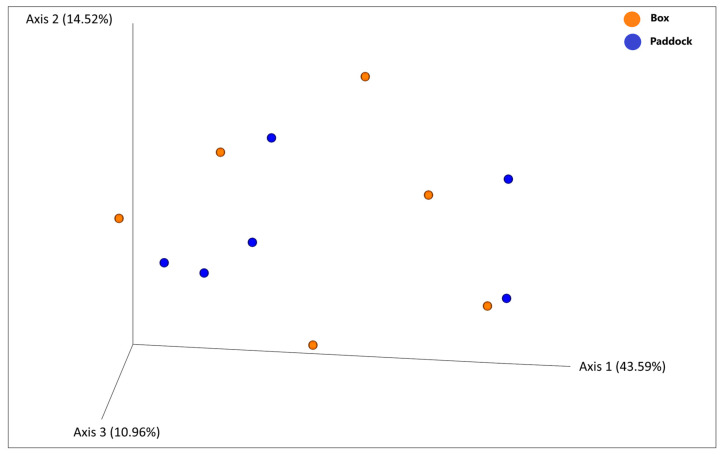
PCoA—Weighted Unifrac.

A beta diversity analysis using PERMANOVA was conducted to explore variations in microbiota composition based on management type. This analysis did not reveal any significant differences between groups categorized by this variable (*p* > 0.05). Principal Coordinates Analysis (PCoA), based on Weighted Unifrac distance matrix, in which Axes 1, 2, and 3 explained 69.07% of the total variability, did not show any distinct patterns of separation in the fecal microbiota composition among the two different groups (Group 1, box and Group 2, paddock) (see Figure 3). PCoA obtained with Unweighted Unifrac, Jaccard, and Bray–Curtis matrices showed similar patterns. This suggests that while there is considerable variability in microbiota composition, it does not significantly differ according to management system.

Finally, the Firmicutes and Bacteroidetes ratio (F/B ratio) was calculated in all fecal samples of horses housed in the two different management systems (Box: C1–C6; Paddock: C7–C12). The results are reported in Table 1. The statistical analysis, conducted with the ANOVA test, did not reveal statistically significant differences between the two groups.

### 3.4. Parasitological Examination

Table 2 shows the results in fecal samples of all the subjects kept in the box (Group 1: C1–C6) and in the paddock (Group 2: C7–C2), measured in EPG (Eggs Per Gram). Most samples display low or no infestation, with 6 out of 12 samples recording 0 EPG. The highest EPG value found is in sample C7 (245 EPG), followed by C6 with 25 EPG. Some samples, i.e., C1 and C2, show minimal levels (10 EPG), while C5 and C12 show even lower values (5 EPG).

## 4. Discussion

In recent years, numerous studies have been conducted to identify markers of welfare and stress in horses [12,17,18]. In addition to its classic assessments, based on behavioral, physiological, and hormonal parameters, increasing attention has been paid to the study of the equine GIT microbiota [39,41,52].

In equines, the composition of GIT microbiota plays a key role in their overall health, significantly influencing nutrient digestion and metabolic processes; modifications to its composition may cause dysbiosis and pathologies [50]. A decrease in diversity and richness of the microbiota, also reported in horse with colic, leads to a decrease in its stability; specific microbial communities such as Lachnospiraceae and Ruminococcaceae may contribute to the maintenance of gut homeostasis and are found to be more present in healthy horses than in horses with colitis [61]. Lachnospiraceae, Ruminococcus, Oscillospiraceae appear to be associated with high performance in racehorses [30,62].

Our investigation showed that the fecal microbiota in the subjects of the two groups considered (Group 1, box and Group 2, paddock), with a hay-based diet, was predominantly composed of Firmicutes (32–53%) and Bacteroidetes (32–47.8%), two phyla that are essential for equine digestive health. These findings align with several other studies, which consistently identify these phyla as the most abundant in the equine gut [20,29,41,44,48,63,64]. Firmicutes, in fact, represent the largest phylum in the equine intestinal microbial community, ranging from 40% up to 90% [39]; Firmicutes and Bacteriodetes represent up to 90% of the gut microbiome in horses [62].

Other notable phyla included Kiritimatiellaeota (formerly Verrucomicrobia) (2.5–12%), highlighted with a higher prevalence in fecal samples of healthy horses in some studies [65].

As for man, dietary intake can significantly influence the intestinal microbial composition; gut microbiota is sensitive to diet, especially consumption of starch, fiber, and fat, and in particular the transition from forage to starch-rich concentrates is associated with rapid changes in fecal microbiota samples [41,66,67]. An increase in Proteobacteria has been observed with high oil and high starch diets [44].

Some studies have described a prevalence of the Bacteroidetes phylum more represented than the Firmicutes in healthy horses and ponies, where horse type and pasture had a significant effect on beta-diversity [29,40]; in contrast, other studies show a predominance of Bacteroidetes in horses with colitis [48]. According to Lara and colleagues [68], more studies are required to establish a causal relationship between these alterations and the occurrence of colic in horses.

In our study, Ruminococcaceae (Firmicutes) is the most abundant family across microbiota samples and within the core ASVs group (31% of total core ASVs). It plays a crucial role in fiber digestion and the production of SCFAs, such as butyrate, which play a protective role in gut health [41].

The data obtained do not seem to show differences in the composition of the fecal microbiota or in the ratio Firmicutes/Bacteriodetes (F/B ratio) concerning the two groups of horses kept under different types of management. Further studies with a larger sample size, also in this breed, will be necessary to confirm the findings of Morrison and colleagues [67] that observed age-related decreases in the F/B ratio, particularly as microbial diversity changes over time [69].

Recent studies suggest that the environmental and managements practices, in addition to the diet, have an impact on GIT microbiota, with horses sharing the same environment having similar GIT microbiota, although the dietary intake is a major factor influencing horse GIT microbiota, with alpha and beta diversity resulting from feeding hay, silage, grass or different type of hay [70]. Management can also impact the GIT microbiota by influencing some risk factors related to the development of colic in horses, such as opening and feeding of a new batch of forage or changing the type of forage and grain or limiting the horse’s daily movement and access to pasture [64]. Some studies conducted on sport horses temporarily kept on pasture suggest the presence of a different composition in the intestinal microbiota compared to horses kept in individual boxes, showing an increase in Ruminococcus and Coprococcus with potential beneficial effects on welfare and also highlighting associations between the composition of the intestinal microbiota and behaviors indicative of poor welfare, such as hypervigilance and withdrawn posture [71].

Furthermore, some management factors involved in horse care, such as prolonged stall housing, can have a negative impact on equine welfare [17]; single box housing can also have a negative effect, leading to the appearance of behavioral abnormalities and stereotypies, as highlighted in observational studies [25,72], which seem to be related to specific alterations in the intestinal microbiota of horses [71].

In relation to management conditions, a significant decrease in alpha diversity and microbiota richness has been observed with increasing age in healthy horses under standard stabling and management systems [40].

The dynamic interaction between intestinal parasites and gut microbiota in horses is not fully understood yet, although young subjects, in particular, are very sensitive to intestinal parasites, especially large and small strongyles, which can be trigger factors for dysbiosis, acute and chronic diseases, including acute larval cyathostominosis, which a mortality rate up to 50% [73]. Some studies in young subjects demonstrate associations with specific compositional alterations of the microbiome, characterized by a lower microbial richness and alterations in the relative abundances of bacterial taxa with immunomodulatory functions that can therefore have an impact on both intestinal absorption and metabolism, in general, and on the development of acquired immunity [74]. Research on parasitic infections and intestinal microbial communities in species of veterinary interest, potentially useful for anthelmintic resistance, seems to be quite limited to date [41,48,74,75,76,77].

Fecal analysis of the subjects in this study showed that most had strongyles EPG 0–25, which generally pose no health risk and do not require treatment unless clinical symptoms appear. No subjects were in the moderate infestation range (EPG 50–200), only one sample presented EPG 245, where an average >1000 EPG can be considered a sign of severe infestation, while values >200 EPG may be regarded as a threshold for treatment [78]. The subject with 245 EPG did not report any alteration of the fecal microbiota compared to the other subjects investigated.

The principal limitations of this study are due to the limited number of animals observed and to the size of the boxes and paddocks used for the management of horses in the farm; in fact, the boxes are rather large in relation to the breed size, permitting, moreover, a visual interaction between the horses, while paddocks of about 400 m^2^ have a quite limited extension. Even the management of the animals housed in boxes, which still had the possibility of spending two hours outdoors in a paddock, may have contributed to the fact that no differences in the composition of the microbiome were detected between the two groups of subjects.

Despite the above limitations, horses of both groups were fed the same diet, and this could be the principal factor that explains the absence of a significant difference in the fecal microbiota examined between the two groups considered.

## 5. Conclusions

The equine gut microbiota is a complex and dynamic ecosystem influenced by factors such as diet, age, environment, and management practices. Understanding these variations is crucial for enhancing equine health and optimizing performance. This knowledge can inform both dietary and management strategies aimed at maintaining a healthy microbiota, thereby reducing the risk of potential health issues. Our findings on the fecal microbiota in purebred Arabian horses contribute to the expanding body of research on the composition of the equine fecal microbiota. Future research should aim to investigate the specific roles of different microbial taxa in equine health and disease, as well as to evaluate the effects of biotic and abiotic factors on microbial diversity. Furthermore, in order to draw more robust conclusions and address the limits of this study, a larger sampling is necessary to provide a more comprehensive understanding of the equine gut microbiota and its role.

## Figures and Tables

**Table 1:** Firmicutes/Bacteroidetes (F/B) ratio in fecal samples in the two management systems (Box: C1–C6); (Paddock: C7–C12).

	Code	F/B Ratio		Code	F/B Ratio
Box	C1	1.03	Paddock	C7	1.14
	C2	1.52		C8	1.00
	C3	0.67		C9	1.02
	C4	1.03		C10	0.76
	C5	0.87		C11	0.84
	C6	0.85		C12	1.21

**Table 2:** Number of strongyles (EPG: Eggs Per Gram) in fecal samples in the two management systems (Group 1, box: C1–C6); (Group 2, paddock: C7–C12).

	Code	Strongyles (EPG)		Code	Strongyles (EPG)
Box	C1	10	Paddock	C7	245
	C2	10		C8	Not detected
	C3	Not detected		C9	Not detected
	C4	Not detected		C10	Not detected
	C5	5		C11	Not detected
	C6	25		C12	5

## Data Availability

The data presented in this study are available on request from the corresponding author.

## Supplementary Materials

The following supporting information can be downloaded at https://www.mdpi.com/article/10.3390/vetsci12040309/s1, Figure S1: Rarefaction curves of alpha diversity approaching the saturation plateau. To the right: each sample with relative color. Figure S2: Relative frequency of bacterial families. Table S1: DATA of the subjects.
