# Supplementary material for: Effect of Management System on Fecal Microbiota in Arabian Horses: Preliminary Results
Source: Vet Sci. 2025 Mar 28;12(4):309. doi: 10.3390/vetsci12040309 (PMC12031164; doi:10.3390/vetsci12040309)
Supplement: Supplementary file 1 [file vetsci-12-00309-s001.zip › Supplementary materials.pdf]

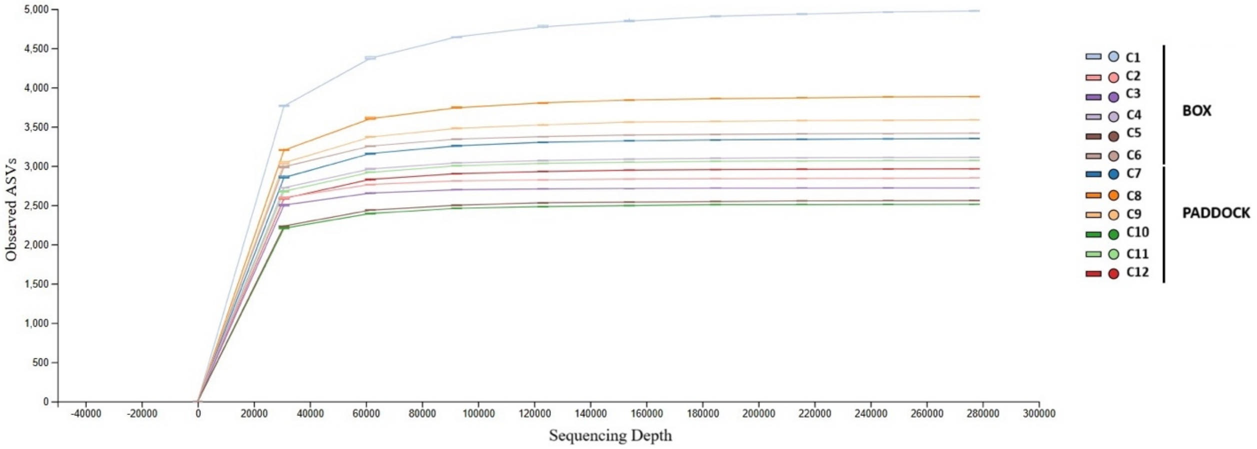

Figure S1. Rarefaction curves of alpha diversity approaching the saturation plateau.
To the right: each sample with relative color

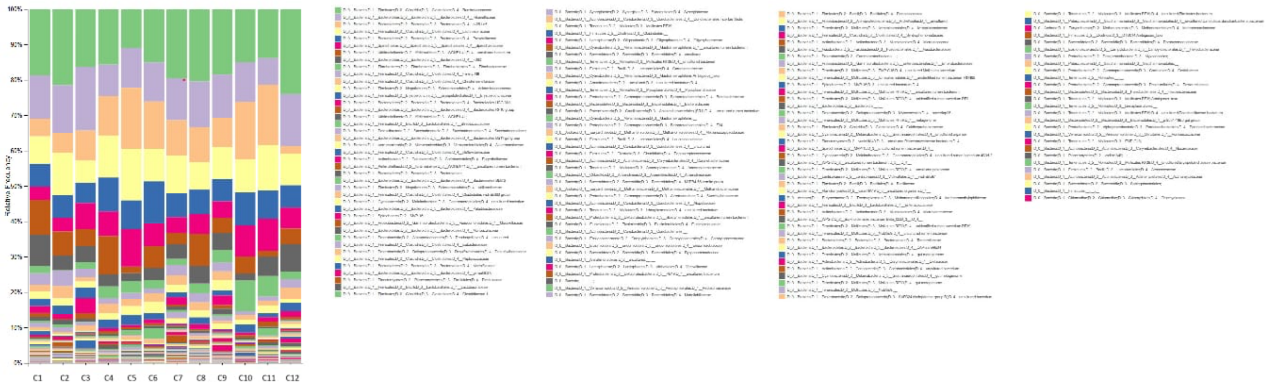

Figure S2. Relative frequency of bacterial families
