# Supplementary material for: Effect of Management System on Fecal Microbiota in Arabian Horses: Preliminary Results
Source: Vet Sci. 2025 Mar 28;12(4):309. doi: 10.3390/vetsci12040309 (PMC12031164; doi:10.3390/vetsci12040309)
Supplement: Supplementary file 1 [file vetsci-12-00309-s001.zip › Table S1.pdf]

Table S1

Code	Management System	Age (years)	Sex
C1	Box	24	F
C2		4	F
C3		6	F
C4		6	F
C5		22	F
C6		2	M
C7	Paddock	21	M
C8		13	F
C9		15	F
C10		9	F
C11		5	F
C12		25	F
